# Hallux Dorsal Curvature of the Distal Phalanx and Its Possible Implications for the Dorsal Osteophyte

**DOI:** 10.3390/medicina60091447

**Published:** 2024-09-04

**Authors:** Emma Guillén Escámez, Martín Redón Martín, Eduardo Nieto-García, Nadia Fernández-Ehrling, Javier Ferrer-Torregrosa

**Affiliations:** 1 Doctorate School, Catholic University of Valencia San Vicente Mártir, 46001 Valencia, Spain; em_chec@mail.ucv.es; 2 Centro Podológico Valencia Martin Redón, 46001 Valencia, Spain; martinredonmartin@gmail.com; 3 Podiatry Department, Faculty of Medicine and Health Sciences, Catholic University of Valencia San Vicente Mártir, C/Ramiro de Maeztu, 14, 46900 Torrent, Spain; eduardo.nieto@ucv.es (E.N.-G.); nadia.fernandez@ucv.es (N.F.-E.)

**Keywords:** dorsal osteophyte, percutaneous foot surgery, pre-surgical goniometric study

## Abstract

*Background and Objective:* The dorsal osteophyte on the distal phalanx of the first toe (hallux) is a reactive bony protrusion that may be associated with pathologies such as onychocryptosis or pincer nail. This study aims to describe and analyze the correlation between three novel measurements—dorsal osteophyte height (HDO), distal phalangeal hyperextension (DPHA), and distal phalangeal curvature (DCDP)—and to evaluate the impact of minimally invasive surgery on the dorsal osteophyte using fluoroscopic data. *Materials and Methods:* A total of 125 fluoroscopic images were analyzed. Baseline measurements for the variables were compared between groups. The key variables included distal phalanx curvature, distal phalanx hyperextension, and dorsal osteophyte height. *Results:* The analysis revealed statistically significant differences in the main group effect for distal phalanx curvature (F [2, 122] = 7.54, *p* < 0.001), distal phalanx hyperextension (F [2, 122] = 28.90, *p* < 0.001), and dorsal osteophyte height (F [2, 122] = 13.64, *p* < 0.001). Significant correlations were found between distal phalanx curvature and distal phalanx hyperextension, as well as between distal phalanx hyperextension and dorsal osteophyte height. However, no significant correlation was observed between distal phalanx curvature and dorsal osteophyte height. *Conclusions*: The findings suggest that minimally invasive dorsal osteophyte surgery effectively restores the distal phalanx to normal conditions, as indicated by the variables studied.

## 1. Introduction

Subungual exostosis of the hallux distal phalanx is a common benign bone tumor formed by mature trabecular bone covered by a fibrocartilage cap [1]. It can be caused by infection or occur secondary to trauma, and it typically has a flattened or dome-shaped morphology [2]. It is more prevalent in women between the ages of 40 and 60 [3].

Clinically, subungual exostosis may be accompanied by other abnormalities such as deformities in the nail layer, yellowish discoloration of the nail layer, or subungual hematoma [4,5,6,7]. Regarding symptoms, patients often experience pain when direct pressure is applied to the nail layer. Diagnosis is usually made through radiology or lateral fluoroscopy of the distal phalanx, and treatment involves surgically removing the dorsal osteophyte.

The dorsal osteophyte, which is sometimes confused with subungual exostosis, does not match Dupuytren’s description from 1847 [2]. It differs from subungual exostosis in its reactive and non-tumoral nature [7]. Additionally, shape differences can be observed in radiological tests. The term “dorsal distal osteophyte” was introduced by R. Baran and colleagues to describe changes in the distal phalanx of the toe associated with pincer-nail [8]. The normal morphology of the distal phalanx of the first toe is longitudinally flat and transversely convex [9].

The upper surface of the distal portion is soft and covered in mesenchyme, forming the nail bed. The dorsal osteophyte appears as a peak-shaped projection in the distal phalanx area, leading to deformities in the nail layer such as pincer-nail or onychocryptosis [10]. While subungual exostosis also has a “peak” or “cauliflower” shape, it appears in the metaphysis of the distal phalanx, causing clinical deformities in the nail bed in the form of subungual nodules [2,3].

The final treatment for both subungual pathologies involves surgical removal of the osteophyte or subungual exostosis, as well as any associated deformities (such as pincer-nail, onychocryptosis, hyperextension of the distal phalanx, etc.). Diagnosis is made using clinical examination, radiology confirmation, and size assessment [11]. Various surgical procedures have been described, including both open surgery [10,12,13,14] and minimally invasive surgery [15,16,17].

With recent advancements in minimally invasive surgery techniques [17,18,19], dorsal osteophyte surgery is performed through a 2–4 mm incision in the distal area of the toe, parallel to the longitudinal axis of the toe, followed by osteophyte removal through drilling [17,20]. While this surgery is generally successful and rarely results in relapses [20,21], there is a lack of anatomical studies that establish a correlation between distal phalanx goniometry and the diagnosis of the pathology. Furthermore, there is a need for classification based on morphometry by measuring pre- and post-surgery changes, similar to what is carried out for other pathologies like Hallux Abductus Valgus (HAV) [22,23,24,25].

Therefore, the main objective of this study is to (a) analyze the relationship between dorsal osteophyte, distal phalanx hyperextension, and distal phalanx curvature in patients diagnosed with a dorsal osteophyte. Based on our hypothesis, we expect a positive correlation between osteophyte height, hyperextension, and distal phalanx curvature. Another purpose of this study is to (b) analyze the effects of minimally invasive surgery for dorsal osteophytes using fluoroscopic evaluation, specifically DCDP, DFHA, and HDO, in affected patients. Finally, our goal is to (c) compare fluoroscopic results (DCDP, DFHA, and HDO) obtained from a non-pathological sample taken after surgery. According to our hypothesis, all fluoroscopic parameters obtained from patients after dorsal osteophyte surgery will be similar to those obtained from non-pathological samples. 

## 2. Materials and Methods

### 2.1. Design

A quasi-experimental pre-/post-surgery study was conducted with a non-equivalent control group.

### 2.2. Participants

A total of 136 fluoroscopic images were obtained from patients at four different Podiatric Clinics (E.Nieto podólogos, Clínica podológica Martín Redón, clínica del peu Podosigma, and Clínica pasito a pasito). All fluoroscopies were performed between July and October 2022.

The eligibility criteria for inclusion were as follows: (a) willingness to accept and sign informed consent, (b) presence of distal pain in the first toe, and (c) adult patients (18 years of age or older).

The exclusion criteria were (a) presence of arthritis/arthrosis, (b) prior HAV surgery, (c) presence of osteoporosis, and (d) history of trauma in the affected area.

Table 1 provides a summary of the participants’ physical characteristics

### 2.3. Procedure

In the initial clinic visit, the patients were informed about the risks and benefits of participating in the research study, including the inherent risks of subungual exostosis surgery, such as recurrence of the deformity, numbness in the toes due to injury of the digital nerves, hematomas, infection of the surgical wound (either superficial or deep, potentially affecting internal structures), undesirable esthetic outcomes such as keloid scars, and perforation of the nail bed, which may lead to the loss of the nail plate, onychodystrophy, or onycholysis. The primary benefit was the elimination of symptoms following the procedure [14]. After signing an informed consent form, the patients underwent evaluation using the Xiscan Serie 4440 Fluoroscopy from Fm Control/Group Alcor (Álava-España). Hallux lateral fluoroscopies were taken, and subjects were categorized as either pathological or non-pathological patients based on the measurements.

Measurements were subsequently taken from patients diagnosed with dorsal osteophyte both before and after surgery. All angle and distance measurements were evaluated three times by the same observer using Osirix MD Software (Pixmeo, Bernex, Switzerland, https://www.osirix-viewer.com/osirix/osirix-md/), and the average of the three measurements was recorded. Two angles and one length were measured.

**Figure 1:**
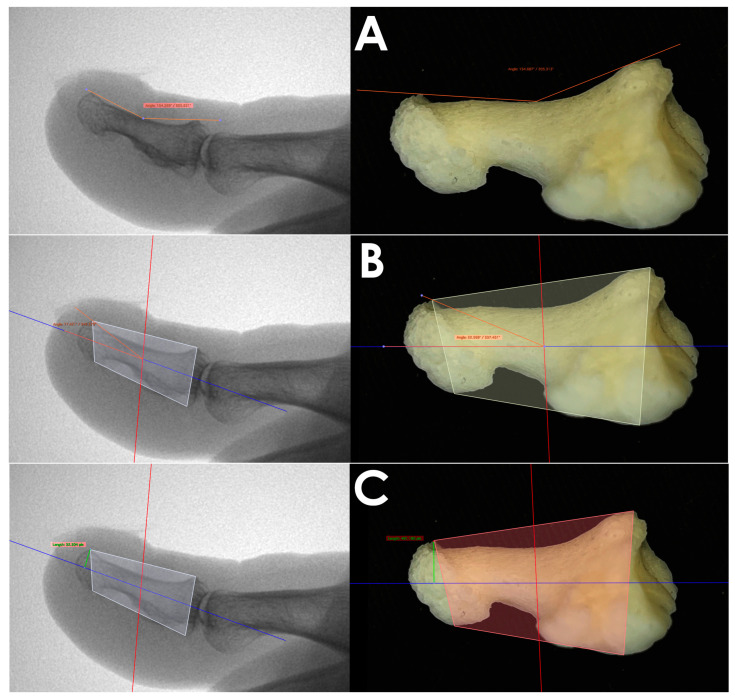
**Left**—Radiological measurements; **right**—Bone imaging of anatomical dissections. Measurements analyzed: (**A**) DCDP, (**B**) DPHA, (**C**) HDO.

The following measurements were taken:

Dorsal curvature of the distal phalanx (DCDP) (orange line): the distance between the highest proximal-dorsal point and the highest distal-dorsal point, as well as the lowest point on the foot sole of the phalanx diaphysis (Figure 1A).

Distal phalanx hyperextension angle (DPHA) (orange line): the angle formed by the mid-axis of the distal phalanx and the highest point of the distal phalanx (Figure 1B).

Height of dorsal osteophyte (HDO) [11] (green line): the distance from the peak of the exostosis at 90° to the mid-axis of the phalanx (Figure 1C).

**Figure 2:**
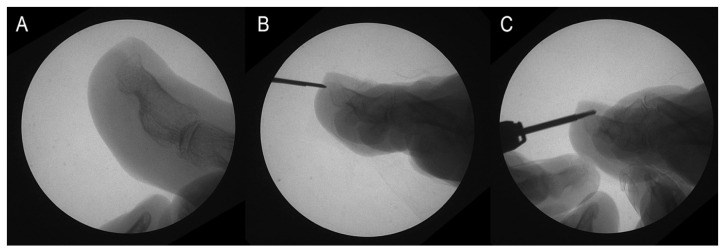
Osteotripsy performance. (**A**) Surgical image. (**B**) Incision with no. 64 Beaver scalper. (**C**) Distal phalax osteotripsy.

Additionally, the podiatrist objectively assessed each patient’s pain and categorized them into two groups: those who reported pain and those who did not. Patients with dorsal osteophytes who reported pain were advised to consider surgery for osteophyte removal during their initial visit (Figure 2A). After receiving comprehensive information about the available treatment options—including surgery and less invasive, non-definitive alternatives such as conservative management with orthotics or nail treatments—patients were given the autonomy to choose the approach they felt was most suitable. Surgery was recommended based on clinical factors such as symptom severity and lack of improvement with conservative treatments. However, some patients chose to avoid surgery due to personal preferences or a preference for less invasive options, a decision that was respected as part of the shared decision-making process with the medical team.

The surgery was performed according to the protocol outlined in [17] during the second visit, following these steps:

1. A 2–4 mm incision was made using a Beaver-64-MIS scalpel on the distal surface wall of the toe, parallel to the longitudinal axis of the toe, followed by drilling of the osteophyte (Figure 2B).
2. The contour of the osteophyte was defined using a blunt elevator to avoid damaging the nailbed.
3. Osteotripsy was performed using medial and lateral movements of a mini-Shannon drill (Figure 2C).
4. Pressure was applied with a surgical spoon to remove the bone paste resulting from the drilling.

Follow-up visits included surgical wound care, data collection, discharge assessments, and post-surgery fluoroscopies. The follow-up period had an average duration of 23 days, ranging from 8 to 30 days. Only one postoperative infection occurred, which was successfully treated with antibiotics, along with two cases of onychodystrophy.

### 2.4. Ethical Statement

The studies involving human participants were reviewed and approved. The study was authorized by the Research Ethics Committee of the Universidad Católica de Valencia San Vicente Mártir, with the registry UCV/2021-2022/201. All the participants were informed about the purpose of the research and were asked to sign a consent form before participating in the study. The procedures were performed in accordance with the ethical standards of the national ethical guidelines for human research ethics and the 2013 revised Declaration of Helsinki. Confidentiality was guaranteed during data collection and subsequent publication of the results.

### 2.5. Statistics Analysis

All variables are presented as mean values and standard deviations (SD). The normal distribution of the data were assessed using the Shapiro–Wilk test (*p* > 0.05), and the assumption of variance homogeneity was evaluated using Levene’s test (*p* > 0.05).

The strength of the relationships between the variables DCDP, DPHA, and HDO was examined using the Pearson correlation coefficient or Spearman’s rank correlation coefficient (for data that did not meet the normality assumption). The strength of associations was interpreted as follows: trivial (0.00–0.09), small (0.10–0.29), moderate (0.30–0.49), large (0.50–0.69), very large (0.70–0.89), nearly perfect (0.90–0.99), and perfect (1.00) [26].

Dependent samples *t*-tests were conducted to evaluate the effects of the surgical procedure. Independent samples *t*-tests were used to compare pathological and non-pathological participants in radiographic variables (DCDP, DPHA, and HDO).

The magnitude of differences was quantified using Cohen’s “d” effect size, with interpretation based on the following scale: trivial (0.00–0.19), small (0.20–0.49), moderate (0.50–0.79), large (0.80–1.19), and very large (>1.20) [26].

Statistical significance was set at *p* < 0.05, and all analyses were performed using statistical software JASP (0.18.3, Amsterdam, The Netherlands).

## 3. Results

A total of 136 participants were initially included in this study. However, 11 patients were excluded as they did not meet the inclusion criteria. The final sample size consisted of 125 participants, with 30 (24%) not showing a dorsal osteophyte and 95 (76%) with a dorsal osteophyte.

Among the 95 patients with a dorsal osteophyte, 35 (37%) decided to undergo treatment. There were no participant dropouts during the surgical procedure. The physical characteristics and descriptive statistics for the variables DCDP, DPHA, and HDO, categorized by patients with or without dorsal osteophyte pathology, are summarized in Table 2.

**Figure 3:**
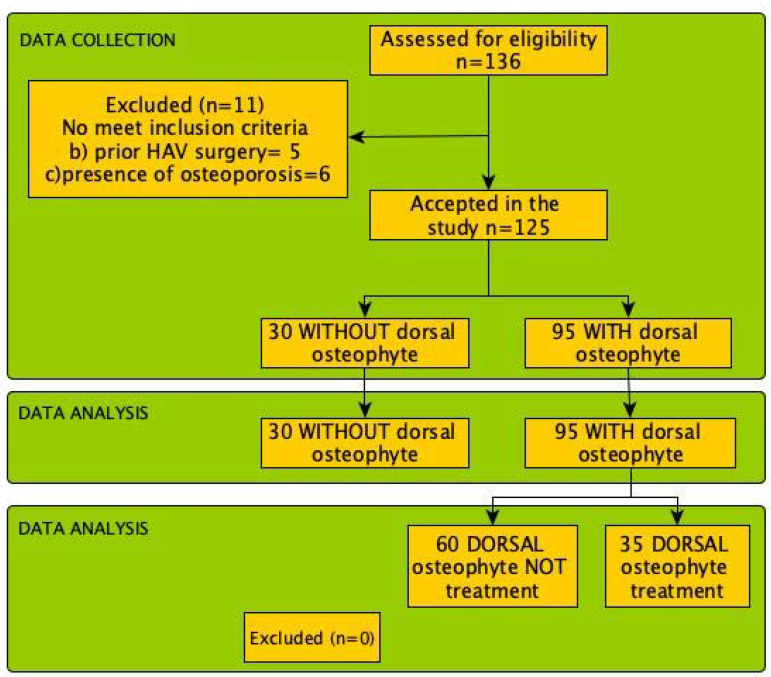
Flow chart participants selection process for the present study.

The selection process of participants for the study is depicted in Figure 3.

During the baseline measurement of the analyzed variables between groups, statistically significant differences were found in the main effect of the group for the variables DCDP (F [2, 122] = 7.54, *p* < 0.001), DPHA (F [2, 122] = 28.90, *p* < 0.001), and HDO (F [2, 122] = 13.64, *p* < 0.001).

For the DCDP variable, the Bonferroni post hoc test revealed statistically significant differences. There was a mean difference (DM) and a 95% confidence interval (CI) of 7° (2.18–11.83), *p* = 0.002, and 7.94° (2.57–13.33), *p* = 0.002, between non-pathological and pathological patients not undergoing surgery and between pathological patients undergoing surgery, respectively.

No statistically significant differences were found between pathological patients not undergoing surgery and pathological patients undergoing surgery (DM [CI95%] = 0.95° [−3.65–5.54], *p* = 1.000).

For the DPHA variable, the Bonferroni post hoc test showed statistically significant differences. There was a DM (CI95%) of −5° (−7.22–−3.78), *p* < 0.001, and −4.13° (−6.05–−2.21), *p* < 0.001, between non-pathological patients and pathological patients not undergoing surgery and between pathological patients undergoing surgery, respectively.

No statistically significant differences were found when comparing pathological patients not undergoing surgery and pathological patients undergoing surgery (DM [CI95%] = 1.37° [−0.27–3.01], *p* = 0.151).

For the HDO variable, the Bonferroni post hoc test showed statistically significant differences. There was a DM (CI95%) of −12.07 pix (−19.35–−4.79), *p* < 0.001, and −17.48 pix (−25.59–−9.37), *p* < 0.001, between non-pathological patients and pathological patients not undergoing surgery and between pathological patients and pathological patients undergoing surgery, respectively.

No statistically significant differences were found when comparing pathological patients not undergoing surgery and pathological patients undergoing surgery (DM [CI95%] = −5.42 [−12.35–1.52], *p* = 0.199).

**Figure 4:**
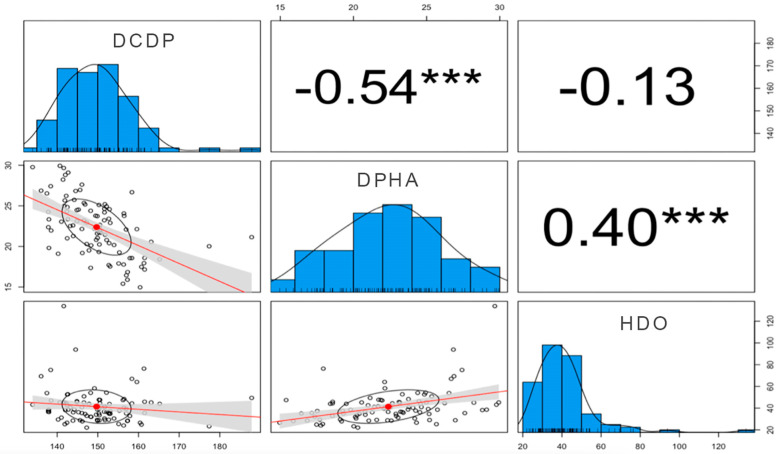
Relationships between studied variables. Correlation between the variables DCDP, DPHA and HDO. The histograms on the diagonal show the distribution of each variable, indicating that DCDP has a relatively normal distribution, while HDO is skewed to the right. Pearson’s correlation coefficients are shown in the upper right of the matrix. *** indicates statistical significance at *p* < 0.001.

Figure 4 summarizes the existing relationships between the variables.

### 3.1. Surgery Evaluation (Pre-Post Surgery)

**Figure 5:**
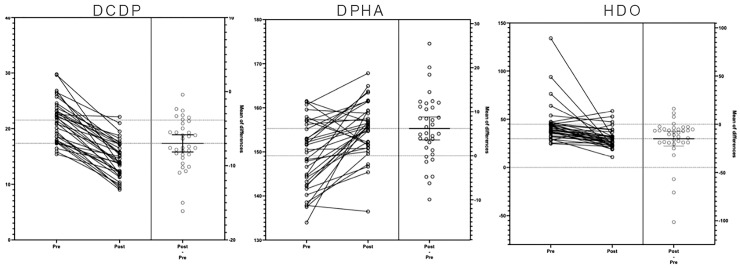
Values pre- and post-surgery for the variables DCDP, DPHA, and HDO. Also shows measures differences and confidence ranges at 90% for each comparison.

T-tests of the dependent samples revealed statistically significant differences in the variables DCDP (t (34) = 4.86, *p* < 0.001, ES = 0.82), DPHA (t (34) = 12.68, *p* < 0.001, ES = 2.07), and HDO (t (34) = 4.00, *p* < 0.001, ES = 0.68) after the surgical procedure. Figure 5 illustrates the measurement differences and 95% confidence intervals.

### 3.2. Comparison between Post-Surgery and Non-Pathological Samples

**Figure 6:**
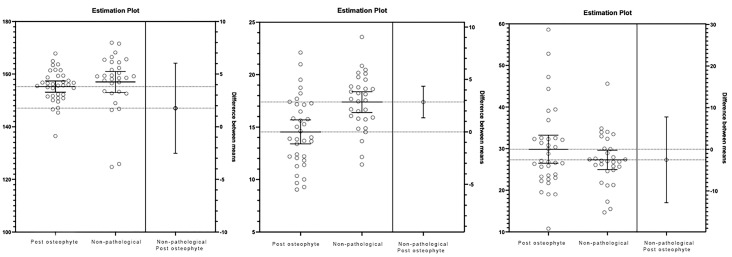
Values pre- and post-surgery for the variables DCDP, DPHA, and HDO. Also shows measures difference and confidence ranges at 95% for each comparison.

On the other hand, *t*-tests of the independent samples did not show statistically significant differences between measurements from the post-surgery group of pathological patients undergoing surgery and the group of non-pathological patients for the variables DCDP (t (63) = 0.825, *p* = 0.413) and HDO (t (63) = 1.227, *p* = 0.224). However, statistically significant differences were observed for DPHA (t (63) = 0.825, *p* = 0.413). The mean difference was 2.84 ± 0.76, with a 95% confidence interval of 1.34–4.32, in favor of the non-pathological group. Figure 6 shows the mean difference and 95% confidence intervals for these comparisons.

No other incidents were reported, and all patients were discharged 10 to 15 days after the surgery.

## 4. Discussion

Upon examining the characteristics of our sample, we observed that a dorsal osteophyte deformity is more prevalent in women (n = 96) than in men (n = 26). These results are consistent with another study on this pathology. However, the average age of our participants (50 to 60 years) is comparable to similar pathologies such as subungual exostosis [3], which is quite different from the average reported in the only reference found about dorsal osteophytes, and Dabrowski’s study suggests that it first appears around the age of 30 [10].

As mentioned earlier, the main objective of this study was to analyze the relationships among DCDP, DPHA, and HDO. The results indicated statistically significant relationships between DCDP and DPHA, as well as between DPHA and HDO. However, no statistically significant relationship was found between DCDP and HDO. Nonetheless, the effect size was considered “moderate” for all variables, suggesting that these structures are related due to their anatomical location.

A study by Noh et al. [11] investigates the association between the height of dorsal osteophyte and nail width for diagnostic purposes. However, their study does not analyze the interrelation of distal phalanx curvature angulation, as we have carried out in the present study.

On the other hand, the objective of this study was also to analyze the effects of dorsal osteophyte surgery on fluoroscopic variables.

The minimally invasive surgical technique applied [17] avoids the complications associated with open surgery, as described in [10,14], including a postoperative nail deformity (onychodystrophy), recurrence and postoperative infections, chronic regional pain syndrome and other reported problems such as postoperative hematomas, prolonged scarring and skin necrosis, and keloids.

After the minimally invasive surgery, all of the patients experienced clinical improvement and there was only one postoperative infection.

In this study, we analyzed the correlation between three measurements: height of dorsal osteophyte (HDO), hyper-extension of distal phalanx (DPHA), and distal phalanx curvature (DCDP). We evaluated their influence on distal phalanx pathology and the results obtained after the MIS procedure. It can be concluded that all variables showed statistically significant improvement with a significant effect size.

Finally, comparing data from patients with a diagnosed pathology who underwent surgery with data from patients without the pathology did not reveal statistically significant differences in the DCDP and HDO variables. Both the angulations and dorsal osteophyte height were restored to non-pathological values. However, differences in DPHA values were observed, indicating that this variable was the most modified. This suggests the idea of implementing a methodology or “pre-surgical” plan to measure the three variables under study using goniometry before the surgical procedure. The aim would be to generate a correction range that brings the distal phalanx of the first toe to a morphology similar to that of non-pathological or healthy patients, as is the case with other pathologies such as HAV [24,25].

### Limitations

Several limitations should be considered when interpreting the results of this study. Firstly, the design of the present study was classified as quasi-experimental since there was no random distribution in the surgery group.

Due to the methodological nature of this study (i.e., pre-/post-surgery), long-term tracking data to analyze dorsal osteophyte recurrence were not recorded. However, a study by the authors of [27] reported no recurrences after an average tracking time of 27 months.

Another limitation in this study has to do with the evaluation of pain in patients. All patients undergoing surgical procedure had reported pain in dorsal/lateral area to unguinal layer before surgery.

Reported pain was not evaluated using any scale, when it could have been classified with the EVA one-dimensional scale.

## 5. Conclusions

In conclusion, the findings of this study demonstrated statistically significant relationships between DCDP and DPHA, as well as between DPHA and HDO, which can be categorized as “moderate relationships.” However, no statistically significant relationship was found between DCDP and HDO. The study also provided insightful results regarding the effects of fluoroscopy on variables after dorsal osteophyte surgical procedures. The post-surgery data showed statistically significant improvements in all variables with a significant effect size.

## Figures and Tables

**Table 1:** Summary of participants’ physical characteristics.

	Pathology, with Surgery (n = 35)	Pathology, without Surgery (n = 60)	Without Pathology (n = 30)
Age (years)	57.40 ± 12.22	54.15 ± 11.54	64.43 ± 11.04
Sex (Men/Women)	7/28 (20%, 80%)	11/49 (18%, 82%)	11/19 (37%, 63%)
Foot (Right/Left)	17/18 (49%/51%)	28/32 (47%/53%)	15/15 (50%/50%)

**Table 2:** Descriptive statistics (average, standard deviation or percentage) for the variables dorsal curvature of the distal phalanx (DCDP), distal phalanx hyperextension angle (DPHA), and height of dorsal osteophyte (HDO) pre-surgery.

	Pathology, with Surgery (n = 35)	Pathology, without Surgery (n = 60)	Without Pathology (n = 30)
DCDP (degrees)	149 ± 7.74	150 ± 8.96	157 ± 10.76
DPHA (degrees)	21.52 ± 3.74	22.89 ± 3.21	17.39 ± 2.65
HDO (pix)	44.78 ± 20.56	39.37 ± 11.29	27.30 ± 6.21

## Data Availability

The data presented in this study are available upon request to the corresponding author.
